# A systematic analysis of online public engagement with 10 videos on major global health topics involving 229 459 global online viewers

**DOI:** 10.7189/jogh.10.010903

**Published:** 2020-06

**Authors:** Iain H Campbell, Igor Rudan

**Affiliations:** Centre for Global Health, Usher Institute for Informatics and Population Sciences, University of Edinburgh, Edinburgh, Scotland, UK

## Abstract

**Background:**

Online interest in issues specific to global health outside of times of pandemics or other crises is rather limited. To achieve long term global health goals, public support and engagement needs to be fostered on a continual basis. Strategies for capturing the attention of the general public online for the persisting problems outside of emergency situations are poorly defined. There are only a few isolated examples of success. In this study we explored the engagement of the viewers with different global health topics that were provided on public and privately advertised YouTube channels.

**Methods:**

We developed the Massive Open Online Course “Survival: The Story of Global Health”, consisting of 10 educational videos on major global health topics. We conducted two experiments in two separate samples of viewers. The first was based on posting videos on a YouTube channel between August 30 and September 30, 2017 and collecting analytics on the viewership. By June 30, 2019 this approach attracted 41 305 viewers. The second experiment was more controlled and conducted on a private YouTube channel and the videos were advertised to reach a high number of viewers. This attracted 188 154 viewers and we collected data on viewers' behaviour using YouTube Analytics. We investigated the nature of engagement, which was defined by 22 different parameters.

**Results:**

In the first experiment, there were clear differences in all measured parameters of engagement based on the topic of the video. Episodes on pandemics (14 594 views) and human evolution (10 761 views) were clear outliers, while the remaining 8 episodes received between 1110 and 3197 views. In the second experiment, there were several clear differences between the 10 videos in the parameters analysed through YouTube Analytics. Episode 2 on maternal and child health had the highest view rate (18.90%), followed by Episode 7 on international organisations in global health (16.83%). At the bottom of the rank were Episode 6 on ageing and dying (view rate of 13.83%) and Episode 5 on non-communicable diseases (view rate 14.59%).

**Conclusions:**

We determined that 5 main interdependent factors contributed to the success or failure of our global health videos: Responsive content, timing, contribution to public debate, emotional valency and endorsement from an authentic scientific voice with a strong public profile. Several of these factors are also recognised as important in marketing research which may indicate the value of such techniques for use in a global health context. In order to communicate long term sustainable solutions to complex issues in a capricious media landscape focused on transient issues the global health community needs to embrace novel marketing approaches.

Global health topics have the ability to capture the broad attention of the mainstream media and the general public whenever there is an international health or humanitarian crisis. However, the online interest in issues specific to global health outside of such times is rather limited. This is unfortunate considering that public health needs amongst populations in poor countries are continuous. There are numerous global health issues which could benefit from a similar level of international public interest. However, strategies for capturing the attention of the general public online for persisting problems outside of emergency situations are poorly developed. There are only a few isolated examples of success, which are often driven by celebrity endorsement.

There are many challenges and gaps in knowledge on how to raise interest in persisting problems in global health, such as pandemics of malaria, tuberculosis or HIV, issues of maternal and child mortality, poor sanitation and housing, low education, lack of access to health care and many others. These problems cause premature disability and death among billions of people living in poor countries today, or in less developed parts of middle-income countries. They are persisting and require constant attention until they are improved, but such topics are not always attractive to the general public whose attention is drawn to privately owned media through long-established methods for capturing public attention. This is what makes a continuous media presence of global health topics a formidable challenge and carefully designed studies are required to explore if successful strategies could be devised.

In order to begin addressing this problem, we developed the Massive Open Online Course “Survival: The Story of Global Health”, consisting of 10 educational videos on major global health topics. We designed two separate experiments to try to understand the differences in responses of the online viewers to the topics we offered and their motivation to engage with those topics. To our knowledge, this is the first such systematic analysis in global health, especially based on a very large sample size. We hypothesized that there will be marked differences between the online viewers' reception of the 10 videos which would mainly be driven by the diversity of topics addressed.

## MATERIALS AND METHODS

We developed the Massive Open Online Course “Survival: The Story of Global Health”, consisting of 10 educational videos on major global health topics. The topics were: (i) survival of the human race; (ii) maternal and child health; (iii) communicable diseases; (iv) epidemics and pandemics; (v) non-communicable diseases; (vi) ageing and dying; (vii) international organizations involved in global health; (viii) UN's Millennium Development Goals and Sustainable Development Goals; (ix) Equity and Inequality; (x) the future of global health and development. These episodes varied in duration from 7 minutes 58 seconds to 19 minutes 50 seconds, with the majority lasting between 10 and 15 minutes.

Once we finished these documentary videos, we conducted the experiment in two separate samples of viewers. This allowed us to investigate differences in engagement of online global health videos when advertised or not. Hence, the first experiment was based on views from a public channel, while the second was advertised to potential viewers through a private channel.

The first experiment was based on creating a YouTube channel named “Survival: The Story of Global Health” between August 30 and September 30, 2017 and allowing it accumulate viewers through organic audience growth and social transmission without advertisement. By June 30, 2019 this approach attracted 41 305 viewers. The second experiment was more controlled and conducted within a YouTube channel which wasn't public and the videos were advertised to potential viewers. This attracted 188 154 viewers and captured viewers' behaviour using YouTube Analytics.

We investigated the nature of engagement, which was defined by 22 different parameters. The parameters included the following: (i) viewers’ age; (ii) viewers' gender; (iii) viewers' income; (iv) viewers' parental status; (v) number of views; (vi) number of “likes”; (vii) number of “dislikes”; (viii-xii) income percentile of the population (5 strata); (xiii) view rate; (xiv) average view duration (as an absolute value); (xv) average view duration (as a proportion of the total duration of the video); (xvi) clicks to find out more about the video; (xvii-xxi) retention rate (4 strata); (xxii) viewers who watched for at least 10 seconds. Based on these parameters, we were able to explore differences in behaviour of the viewers across the 10 videos, assuming that the diversity of topics would necessarily lead to notable differences between the 10 videos.

## RESULTS

The results of the first experiment are shown in **Table 1**. The table shows the number of views, likes, dislikes and the rate of likes per views that resulted from posting 10 videos on a YouTube channel called “Survival: The Story of Global Health”. Over a period of just under 2 years all videos together attracted 41 305 views. There were clear and substantial differences in interest in different topics, which were more than 10-fold. The most popular video, Episode 4 on epidemics and pandemics, attracted 14 594 views, followed by Episode 1 which attracted 10 761 views. At the opposite end, Episode 6 on ageing and dying attracted 1110 views and Episode 10 on the future of global health and development attracted 1375 views. The other 6 episodes ranged between 1541 and 3117 views, showing that Episodes 1 and 4 were real outliers in terms of generating public interest.

**Table 1:** Number of views, likes, dislikes and the rate of likes per views that resulted from posting 10 videos on a YouTube channel called “Survival: The Story of Global Health”

	Views	Likes	Dislikes	Likes per views
Episode 1	10 761	101	0	0.93%
Episode 2	3117	26	1	0.83%
Episode 3	2793	31	0	1.11%
Episode 4	14 594	80	3	0.55%
Episode 5	1600	20	1	1.25%
Episode 6	1110	24	0	2.16%
Episode 7	1541	13	0	0.84%
Episode 8	1988	16	0	0.80%
Episode 9	2426	14	0	0.58%
Episode 10	1375	23	0	1.67%

The analysis of “likes” and “dislikes” showed that topics of general public interest (pandemics, human history) were far more likely to attract likes than videos about internal global health debates and policy, which is not surprising. In 41 305 views, we only recorded five dislikes. Three of them were related to Episode 4. Since this episode was presented in the context of promoting the value and importance of vaccines, it is quite possible that those rare dislikes were left by viewers who supported antivaxxer views - ie, groups of people who question the value of vaccines. **Table 1** shows both - the total number of likes and the rate of likes per views. According to this parameter, the most actively liked was Episode 6 on ageing and dying, whose rate of likes per views was 2.16%. It was followed by Episode 10 on the future of global health and development with a rate of 1.67% and Episode 5 on non-communicable diseases with 1.25%. At the other end, Episode 4 on epidemics and pandemics had a rate of 0.55%, while Episode 9 on inequality and equity had 0.58%. Other episodes ranged between 0.80% and 1.11%. This analysis shows that there were marked differences in the rate of likes per views and that the videos which were watched the least seemed to have the highest rates of likes per views.

The results of the second experiment are shown in **Table 2****,** **Table 3** and **Table 4****.** Despite the relative uniformity of results driven by mass audience behaviour there were some marked differences between the 10 videos in the parameters analysed through YouTube Analytics. The same budget was allocated to each episode and we ensured the same settings were applied for each run of the advert for each of the 10 videos. Still, the YouTube algorithms assigned views differently to the 10 videos, leading to substantial differences in the initial number of views, albeit less striking than those observed in the first experiment. The difference here between the most and the least viewed videos was only 2-fold, while in the first experiment they were 13-fold. The most viewed was Episode 3 on communicable diseases with 31 026 views and the least viewed was Episode 8 on the UN's Millennium Development Goals and Sustainable Development Goals with 13 984 views. As these differences arose entirely from the allocation and advertising campaign performed by YouTube algorithms, we cannot explain why these differences arose. Still, it is reassuring that the majority of videos received between 14506 and 20388 views and they were quite comparable in that sense.

**Table 2:** YouTube Analytics results of the experiment conducted within the closed YouTube channel where the videos were advertised to potential viewers

	Views (started viewing)	Views (at least 10 s)	View rate (topic of interest?)	Average view duration (minutes)	Average view duration (%)	Clicks (“find out more”)	Retention (percentage watching at 25%)	Retention (percentage watching at 50%)	Retention (percentage watching at 75%)	Retention (percentage watching at 100%)
Episode 1	19 614	3183	16.23%	3.14	35.1	62	6	4	3	3
Episode 2	20 388	3854	18.90%	3.28	43.3	74	9	6	5	4
Episode 3	31 026	4922	15.86%	4.55	32.0	114	5	4	3	3
Episode 4	17 351	2552	14.71%	6.00	30.2	47	4	3	3	2
Episode 5	25 367	3702	14.59%	4.37	31.4	95	5	3	3	2
Episode 6	14 506	2006	13.83%	5.15	33.5	46	5	4	3	3
Episode 7	14 718	2477	16.83%	3.27	39.9	45	7	5	4	3
Episode 8	13 984	2171	15.52%	3.41	41.6	49	7	5	4	3
Episode 9	16 499	2439	14.78%	3.58	43.2	56	5	5	4	4
Episode 10	14 701	2351	15.99%	4.45	35.3	55	6	4	3	3

**Table 3:** YouTube Analytics results of the experiment conducted within the closed YouTube channel where the videos were advertised to potential viewers by age and gender

	% aged 18-24	% aged 25-34	% aged 35-44	% aged 45-54	% aged 55-64	% aged 65+	% unknown age	% female	% male	% unknown gender
Episode 1	23.97	28.36	17.24	9.42	5.65	3.29	12.03	32.95	57.96	9.07
Episode 2	24.10	27.86	17.90	8.82	5.52	3.34	12.42	31.44	58.48	10.06
Episode 3	25.07	27.71	18.00	8.63	5.85	3.57	11.15	37.60	54.38	8.00
Episode 4	28.95	28.09	15.43	10.18	5.01	3.56	8.73	34.87	60.93	4.19
Episode 5	25.25	27.74	16.26	10.02	5.07	2.99	12.64	36.11	54.64	9.23
Episode 6	25.12	29.36	16.90	9.52	5.43	3.04	10.56	34.84	58.62	6.53
Episode 7	27.41	27.33	16.39	9.52	6.09	4.11	9.12	34.07	59.50	6.41
Episode 8	25.33	27.17	19.02	9.07	5.29	3.86	10.22	38.73	54.53	6.72
Episode 9	25.33	27.92	17.63	9.84	6.43	3.32	9.51	33.94	59.65	6.39
Episode 10	25.35	29.26	16.75	8.12	6.38	3.19	10.93	35.68	57.29	7.01

**Table 4:** YouTube Analytics results of the experiment conducted within the closed YouTube channel where the videos were advertised to potential viewers by income status and parental status

	% unknown income	% in bottom 50% income	% in 41%-50% income	% in 31%-40% income	% in 21%-30%	% in 11%-20%	% in top 10%	% parent status	% not parent status	% unknown parent status
Episode 1	69.14	8.79	1.91	2.76	2.79	4.24	10.33	18.34	53.75	27.89
Episode 2	68.73	9.13	2.10	2.90	3.34	4.22	9.54	17.56	53.99	28.43
Episode 3	63.20	14.05	2.60	3.61	3.94	4.67	7.90	25.17	59.04	25.17
Episode 4	78.20	8.43	1.56	2.23	2.01	2.82	5.97	12.57	67.71	19.71
Episode 5	64.70	13.52	2.43	3.05	3.51	4.80	7.91	15.61	58.31	26.06
Episode 6	62.26	13.16	2.24	3.09	4.03	5.03	10.16	15.70	61.06	23.23
Episode 7	60.59	14.85	2.13	3.55	3.35	5.24	10.25	15.26	62.41	22.32
Episode 8	78.30	8.79	1.79	2.30	2.21	2.62	3.96	16.81	58.22	24.96
Episode 9	58.42	15.53	2.58	3.73	3.97	5.24	10.49	15.25	62.68	22.05
Episode 10	63.29	14.37	2.29	2.84	3.82	5.10	8.25	16.88	59.37	23.73

The most interesting question of this experiment was the result shown in the 3^rd^ column of **Table 3**, ie, the view rate for each video. This parameter was derived as the number of views that lasted beyond 10 seconds divided by the total number of views. Given that in the first 10 seconds of each of the 10 videos the title of the episode was displayed, using the same design in the background, this column is a direct indication of the interest of a larger number of diverse online viewers in the topic covered by each video. There were, again, marked differences in view rates. The video capturing the largest audience was Episode 2 on maternal and child health (with a view rate of 18.90%), followed by Episode 7 on international organisations in global health (view rate of 16.83%). At the bottom of the rank were Episode 6 on ageing and dying (view rate of 13.83%) and Episode 5 on non-communicable diseases (view rate 14.59%).

The next parameter we studied was average view duration. This parameter is interesting and useful because it is neither affected by the number of views that each video received nor the duration of the video, which makes it fully comparable across the 10 videos. The topic that retained the audience for the longest time was Epidode 4 on epidemics and pandemics (6.00 minutes), followed by Episode 6 on ageing and dying (5.15 minutes) and Episode 3 on communicable diseases (4.55 minutes). Episode 1 on human population survival, Episode 2 on maternal and child health and Episode 7 on international organizations in global health had the shortest retention of viewers, ranging from 3.14 to 3.27 minutes. Clicks to find out more about each video ranged from a maximum of 114 for Episode 3 on communicable diseases, which was a real positive outlier, to only 45-49 for Episodes 4, 6, 7 and 8.

The proportion of the viewers still watching the video at 25%, 50%, 75% and 100% is a very useful indicator. It shows that the drop-off rate for the advertised videos on YouTube is very high. This is the case for most YouTube campaigns as viewers generally use YouTube for entertainment and not the adverts. Typically as many as 91%-96% of viewers would drop off each video by 25% of its total playtime. Episode 2 on maternal and child health seemed to achieve slightly higher retention than other episodes. This shows that it is remarkably difficult to convert advertised videos on global health into committed views and a very large number of showings will be required to achieve substantive audiences in global health themes. However, it is worth noting that 100% completion rates ranged between 2% and 4%, which is not an unreasonable result for an entirely untargeted YouTube campaign, and may represent one of the most useful results of this study.

The findings in **Table 3** and **Table 4** show the demographics of the viewers who were most likely to engage with the content. A careful examination of the differences between the 10 episodes in terms of viewers' gender and age (**Table 3**), income brackets and parental status (**Table 4**) did not reveal any appreciable differences. Even the observed slight differences in income brackets may not be real as they may be biased by a rather high proportion of those with unknown income.

## DISCUSSION

YouTube advertising works on the basis of showing a large number of low-cost adverts to targeted audiences and achieving a response from small percentages. The percentage of people watching beyond ten seconds of the video is generally quite small (around 27.7% on average) even with targeted commercial content. However the low cost per view (US$0.044 on average) allows the ads to be shown thousands of times without incurring large costs [1]. Click-through rates are generally even smaller since the advert is interrupting video content the user intended to watch. With precise audience targeting these numbers can be increased and by showing the ads at scale large audiences can be reached.

While the first experiment was entirely unaffected by any form of advertising, our second experiment launched untargeted video adverts. This design aimed to attribute improvements in any given metric mostly to the content of the video. This approach allowed us to reach a general audience who would not be biased for or against watching global health content.

### The impact of responsive content, timing and public debate

The first experiment showed Episode 4 on pandemics and epidemics to be a clear outlier in popularity with 14 504 views. It was quite easy to discern the reason for this result as the episode triggered a large public debate in Croatia; the home-country of our narrator (IR), which at the time was experiencing a measles outbreak [2]. Many of the large Croatian news broadcasters, online newspapers and public figures began to share the video and an intense public debate was triggered. As the measles outbreak was an issue of concern in Croatia at the time, the video's addressing of the wider historical issue of pandemics served to focus the public debate in this particular crisis towards the value of vaccination. As a result of this reaction to the video the narrator was invited to give several interviews in Croatian media. The full “Survival: The Story of Global Health” documentary series was then shown as a primetime show on Croatian National Television's Channel 1 and reached around 1 million cumulative viewers over the course of the series.

Global health research is often focused on long-term development goals and as a result of this global health media content does not always feature prominently in the day-to-day media. However, there have been several examples of the global health community successfully using the media attention generated by extreme events, such as pandemics, to drive changes in public opinion, infrastructure and policy reform. The success of Episode 4 highlights the importance of releasing relevant media content precisely at such points of a public concern and an ongoing debate.

The commercial advertising industry is familiar with the value of such approach to engage with the public. As the pace of social media sharing accelerates, many marketing agencies have developed a form of reactive marketing that is sometimes referred to as “Piggyback Marketing” [3]. This term refers to marketing which redirects attention from existing traffic sources such as popular news stories or debates in the media. In this case the measles epidemic was already a popular story in Croatian media and our video served to refocus the public debate towards the value of vaccines. This kind of reactive content can be used to shift the discussion towards scientific evidence and long-term sustainable solutions. Much of the online debate in Croatia focused on perceived short-term dangers of vaccination which were not based on scientific evidence. Our video provided a wider scientific and historical overview of the devastating impact of pandemics on a longer time scale and therefore indirectly promoted the uptake of vaccines.

It is interesting to note that while this episode was the most popular in terms of views and the 2^nd^ most popular in terms of likes, it also received a number of negative comments in the “comments” sections of online newspapers, as well as the highest number of dislikes on YouTube – albeit only three in total. This seems to indicate that controversial issues which generate strong views on either side of the debate may be areas where greater impact on public opinion can be achieved. In the debate on vaccination we made a positive impact by simply presenting unbiased scientific and historical information in an appealing video format that was easy to follow.

### Negative and positive emotional valence

In both experiments episode 6 generated the least total engagement from the audience. In the first experiment it received the smallest number of views (n = 1110). In the second experiment it received the lowest view rate (13.83%) and the second lowest number of clicks (n = 46). The view rate shows how many people kept watching after 10 seconds, which is approximately the time that the title of the episode remains on screen. The low number of views and high drop-off rate after viewing the title screen may be due to the particularly negative emotional valency of the episode title in comparison to the aspirational/positive titles of many of the other episodes. “Episode 6: Are Ageing and Dying Inevitable?” may have been a hard-sell for people casually looking for entertainment on YouTube. This observation is in line with positive marketing philosophy as delineated by Berger [4] who noted that positive content is more likely to become viral than negative content. However, it should also be noted that this episode received the highest likes-per-view ratio in the first experiment and the second highest view duration in the second experiment. This indicates that those who did decide to watch the episode were highly engaged with it. Berger notes that negative sentiment content can still be highly engaging if it leads to high emotional arousal.

The serious and sometimes tragic subject matters that global health researchers need to convey to the public are far more difficult to communicate on a popular level than many other fields of research. This is due to their seriousness and often “negative” emotional valency. When the goal is to raise public awareness of such issues, it may be beneficial in some cases to present these topics with different kinds of emotional stance. The increasing recognition that the public have become fatigued by negative charity advertising was catalysed by research from Birbeck and the London School of Economics [5]. They concluded that public perception of international aid agencies has become increasingly negative due to resentment of “excessively traumatic campaigns”. While negative messaging can drive short-term donations and support, there are indications that this is not a sustainable strategy. The implication is that charities need to build long-term relationships with supporters based on positive emotional experience. Referring to such short term negative advertising strategies, co-founder of “Regarding Humanity”, Linda Raftree, said: “We know that organisations need to raise funds for their work, but when it comes to such advertising and campaign imagery, they’re often acting detrimentally to their long-term goals” [6]. An excellent creative example of a successful campaign focusing on a positive approach to difficult issues was the Sick Kids Foundation advertising which presented sick children and doctors as super-heroes fighting against illness and disease [7]. The campaign raised significant financial support for the hospital and improved morale among the staff.

### The impact of public figures

In the first experiment, the view count was greatly helped by the already growing public profile of the narrator (IR), who presented the series, as a “public intellectual” in Croatian media space. The news that the narrator was launching a new video series generated a high number of views on video 1 (n = 10 761), but this dropped to 3117 and 2793 on episodes 2 and 3, respectively. The surge in viewership in Episode 4 after the drop-off indicates that the popularity of Episode 4 cannot be solely attributed to the narrator's public profile, but rather the content of the video. The combination of a “well known public intellectual” figure with a timely contribution to an impassioned public debate seems to be an effective combination to achieve viral spread.

“Celebrity” status can clearly bring attention to global health subjects. However, Markham [8] provides evidence that members of the public who are interested in “celebrity culture” are not necessarily the most likely to participate in supporting important social issues. For this reason we believe that some of the most effective “celebrity figures” to promote global health topics will not be “traditional celebrities” - who may be famous for a variety of interesting reasons - but rather scientists and researchers who have stepped into public space. A clear example was the late Dr Hans Rosling, who brought authenticity to the public debate and had the authority of a scientist and a professional researcher. We believe this was a key feature of the narrator's appeal to the Croatian public, given that he is well recognised in the Croatian media as one of the few leading international scientists of Croatian origin [9].

## CONCLUSIONS

We concluded that for global health media to communicate long term sustainable solutions to complex issues in a capricious media-landscape it could benefit from adopting some of the strategies currently being used in commercial marketing. These include creating responsive content released with precise timing while interest in the topic of concern is reaching maximum growth. More attention should be given to the emotional tone of global health messaging and long-term engagement with viewers fostered through primarily positive interactions as audiences become fatigued with traditional guilt-based or negative emotional appeals. Finally, our results indicate that the public responds positively to global health messages delivered by a presenter with an authentic scientific background and expertise.

## References

[1] 2017 YouTube advertising benchmarks - Strike Social. Strike Social. 2019. Available: https://strikesocial.com/blog/YouTube-advertising-benchmarks/. Accessed: 28 July 2019.

[2] Igor Rudan. Većina zaboravlja razloge zašto se cijepimo [Internet]. Liberal.hr. 2019. [in Croatian]. Available: https://www.liberal.hr/igor-rudan–vecina-zaboravlja-razloge-zasto-se-cijepimo-667. Accessed: 29 July 2019.

[3] The Positives and Negatives of Piggyback Marketing [Internet]. Medium. 2019 [cited 29 July 2019]. Available: https://medium.com/h2o-creative-communications/the-positives-and-negatives-of-piggyback-marketing-2e49098b8dcd. Accessed: 29 July 2019.

[4] Berger J Milkman K What makes online content viral? J Mark Res. 2012;49:192-205. 10.1509/jmr.10.0353

[5] Seu IB. Caring in crisis? Communications and public reactions to humanitarian crises and international development causes, 2014, Birkbeck, University of London, London, UK.

[6] Meade A. Emotive charity advertising – has the public had enough? The Guardian. 2019. Available: https://www.theguardian.com/voluntary-sector-network/2014/sep/29/poverty-porn-charity-adverts-emotional-fundraising. Accessed: 29 July 2019.

[7] Beltrone G, Beltrone G. Ad of the Day: Sick Kids Are the Ultimate Fighters in Brilliant Hospital Ads by Cossette. Adweek.com. 2019. Available: https://www.adweek.com/brand-marketing/ad-day-sick-kids-are-ultimate-fighters-brilliant-hospital-ads-cossette-174134/. Accessed: 31 July 2019.

[8] Markham T Celebrity advocacy and public engagement: The divergent uses of celebrity. Int J Cult Stud. 2015;18:467-80. 10.1177/1367877914528542

[9] Rudan: Teško mi je kolegama predočiti Hrvatsku kao zemlju u problemu jer je pamte kao raj na Zemlji. [in Croatian]. Vecernji.hr. 2019. Available: https://www.vecernji.hr/techsci/igor-rudan-tesko-mi-je-kolegama-predociti-hrvatsku-kao-zemlju-u-problemu-jer-je-pamte-kao-raj-na-zemlji-1109887. Accessed: 30 July 2019.

